# Optimal synthesis and characterization of Ag nanofluids by electrical explosion of wires in liquids

**DOI:** 10.1186/1556-276X-6-223

**Published:** 2011-03-15

**Authors:** Eun Ju Park, Seung Won Lee, In Cheol Bang, Hyung Wook Park

**Affiliations:** 1 School of Mechanical and Advanced Materials Engineering, UNIST 100 Banyeon-ri, Eonyang-eup, Ulju-gun, Ulsan Metropolitan City 689-798, Republic of Korea; 2 Interdisciplinary School of Green Energy, UNIST, 100 Banyeon-ri, Eonyang-eup, Ulju-gun, Ulsan Metropolitan City 689-798, Republic of Korea

## Abstract

Silver nanoparticles were produced by electrical explosion of wires in liquids with no additive. In this study, we optimized the fabrication method and examined the effects of manufacturing process parameters. Morphology and size of the Ag nanoparticles were determined using transmission electron microscopy and field-emission scanning electron microscopy. Size and zeta potential were analyzed using dynamic light scattering. A response optimization technique showed that optimal conditions were achieved when capacitance was 30 μF, wire length was 38 mm, liquid volume was 500 mL, and the liquid type was deionized water. The average Ag nanoparticle size in water was 118.9 nm and the zeta potential was -42.5 mV. The critical heat flux of the 0.001-vol.% Ag nanofluid was higher than pure water.

## Introduction

As noble metal materials, silver nanoparticles exhibit significantly distinct physical, chemical, and biological properties. Silver nanoparticles have attracted attention in a wide range of application fields [1-4]. Their unique properties result from particles on the nanoscale that are monodispersed and unagglomerated.

Nanofluids, dispersed nanoscale particles suspended in a base fluid [5], have drawn tremendous interest from scientific and industrial communities because of their unique properties. They have been used in many industrial applications such as heat transfer, automotive, electronic, biomedical device manufacturing, and others [6-10]. In particular, nanofluids have gained interest as heat transfer fluids. Due to the high thermal conductivity of nanoscale metal particles, metal-nanofluids may significantly enhance thermal transport capabilities. Nanofluids have shown the most promise as coolants because they enhance critical heat flux [CHF] [11-13].

Nanofluids are produced by two methods: a one-step method and a two-step method. The two-step method forms nanoparticles using physical or chemical synthesis techniques and disperses them in basic fluids. The one-step method forms nanoparticles directly in basic fluids [14-16]. A promising one-step method, a physical synthesis technique, is the electrical explosion of wires in liquids [EEWL]. EEWL has advantages, such as high-purity nanoparticle production without surfactants (non-toxic), in contrast to chemical techniques, oxidation prevention using dense media, and spherical nanoparticle production. The greatest advantages are simple evaporation and condensation, short production times, and possibility of mass production. The EEWL method has been studied by many researchers. Most previous studies were conducted with various materials under gaseous conditions. Table 1 summarizes previous studies on the EEWL method.

**Table 1:** Previous works on electrical explosion of wires

Reference	Material	Condition	Control parameter	Average size
Karioris and Fish [20]	Au, Ag, Al, Cu, Fe, W, Mo, Ni, Th, U, Pt, Mg, Pb, Sn, Ta	Air	Capacitance	30-50 nm
			Applied voltage	
			Wire mass	
			Ambient gas	
Couchman[21]	Al, Ni, Au, Pt	Air		10 nm
Phalen [22]	Ag	Air	Voltage: 0-30 kV	0.3 μm
Cho [23]	Ag	Water		<100 nm
Park [24]	Ag	Water	One-step	88.8 nm
			Two-step	

**Figure 1:**
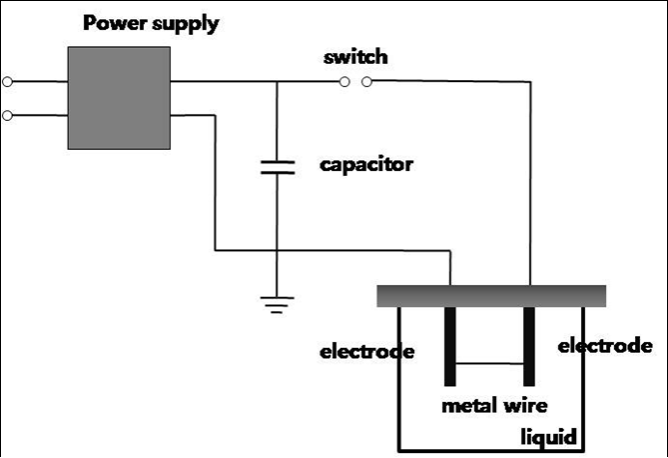
**Schematic diagram of the experimental system for the EEWL process**.

**Figure 2:**
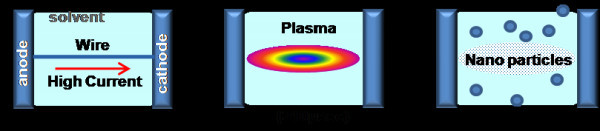
**Production principles for nanoparticles produced by EEWL**.

A schematic illustration of the experimental system is shown in Figure 1. The system consists of a container for liquid media for the explosion process and a simple discharge circuit, which includes a high-voltage power supply, a capacitor, and a spark-gap switch. A metal wire (conductor) was placed in the container filled with the liquids. The capacitor was charged using an applied voltage. The amount of stored energy in the capacitor was *W *= 0.5*CV*^2^, where *C *is the capacitance and *V *is the charged voltage. By closing the switch, the current was allowed to flow through the wire. The current deposited the electrical energy in the wire due to its finite resistance. Thus, the wire located between the two electrodes melted, vaporized, and turned into plasma. Finally, nanoparticles were formed by interaction with the liquid. The vaporized particles were condensed more efficiently in the liquid than in ambient air. The basic principle of the method is illustrated in Figure 2.

Although the formation of nanoparticles by EEWL is complex, including melting, vaporization, and condensation, it can be explained based on the energy deposited in the wire. In the EEWL process, the energy deposited in the wire can be calculated by time integration using the measured voltage and current waveform of the power dissipated in the wire according to the following equation:

where *W *is the energy deposited in the wire, *v *is the voltage, and *i*(*τ*) is the time integration.

Parameters that can influence the properties of particles synthesized by EEWL include electrical circuit parameters (voltage, capacitance, inductance); the amount of energy deposited in the wire; the properties of the exploding wire (diameter, length, defects); sublimation of the metal; and properties of the liquid (viscosity, thermal conductivity, breakdown strength).

In this study, we produced and characterized pure Ag nanofluids by EEWL. We examined the energy deposition in the wire under various conditions and focused on controlled particle size and stability. To identify the effects of key parameters in EEWL, we designed the experiments using Minitab and observed the Ag particle size, morphology, and dispersibility in nanofluids. For applications such as cooling system for electronics and nuclear reactors, it is important to increase the CHF. Thus, to determine potential for increased CHF, we used the pool boiling test of the Ag nanofluids. Finally, to decrease the particle size and improve the dispersibility of Ag nanofluids, we optimized the processing parameters for EEWL using a response optimization technique [ROT].

## Experimental setup

The Ag wire (0.1 mm in diameter) was installed in the cylinder filled with the liquids. The capacitor was charged to 3 kV and the current flowed through the wire when the spark-gap switch was closed. High-temperature plasma was generated by the electrical energy deposited in the wire and was condensed by the basic fluids. A self-integrated Rogowsky coil and a high-voltage probe were used to measure the current and voltage waveforms, respectively.

Many parameters can influence the particles produced by EEWL. In this work, we examined the effects of capacitance, wire length, and liquid type and volume. The experimental parameters are summarized in Table 2.

**Table 2:** Summary of experimental parameters

Capacitance	7.5 μF, 30 μF
Charging voltage	3 kV
Material	Silver
Wire diameter	0.1 mm
Wire length	2.8 mm, 3.8 mm
Liquid	Deionized water, Ethanol
Liquid volume	500 mL, 1,000 mL
Ag concentration	0.001 vol.%

We analyzed the effects of the energy deposited in the exploding wire on the size and shape of the Ag nanoparticles. The morphology was observed by high-resolution transmission electron microscopy [TEM]. The size and zeta potential of the nanoparticles were measured using a submicron size and zeta potential measuring system (Nano ZS, Malvern, Worcestershire WR14 1XZUK, UK). Adsorption spectra were analyzed using UV/Vis spectroscopy. Design of experiments [DOE] was performed to optimize the control factors for EEWL.

## Results

### Manufacturing of Ag nanofluids

**Figure 3:**
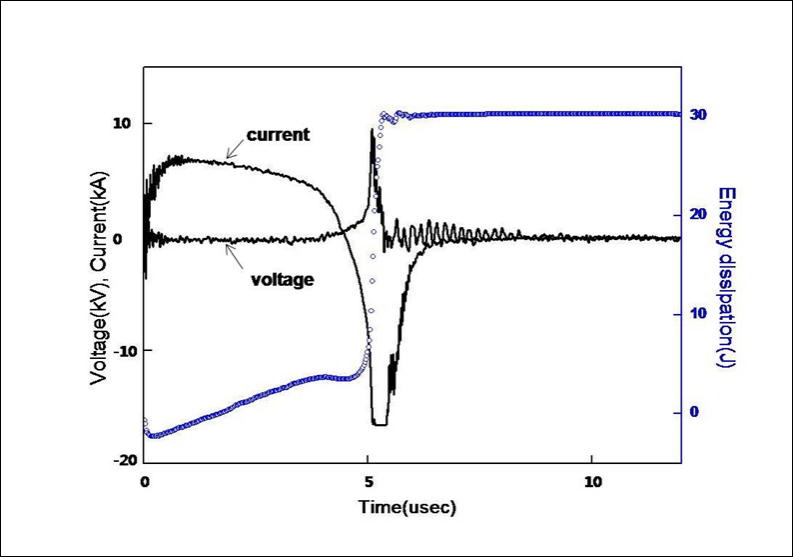
**Voltage, current, and energy deposited in the wire during the explosion process**.

Figure 3 shows the current and voltage waveform measurements. When the capacitor was discharged, the current decreased, and after a certain time, a sudden current drop and sharp rise in voltage occurred. The current drop and voltage rise resulted from an increase in the resistivity of the wire due to vaporization. The resistance increased several orders of magnitude, and the current in the circuit was interrupted. The inductive energy stored in the stray inductance of the circuit generated a high voltage, which was higher than the initial voltage of the capacitor. Once vaporization occurred, an arc discharge formed between the electrodes, and the current flowed through the low-resistance arc plasma. Thus, the wire was heated and vaporized by the energy deposited in the wire until the arc discharged.

**Figure 4:**
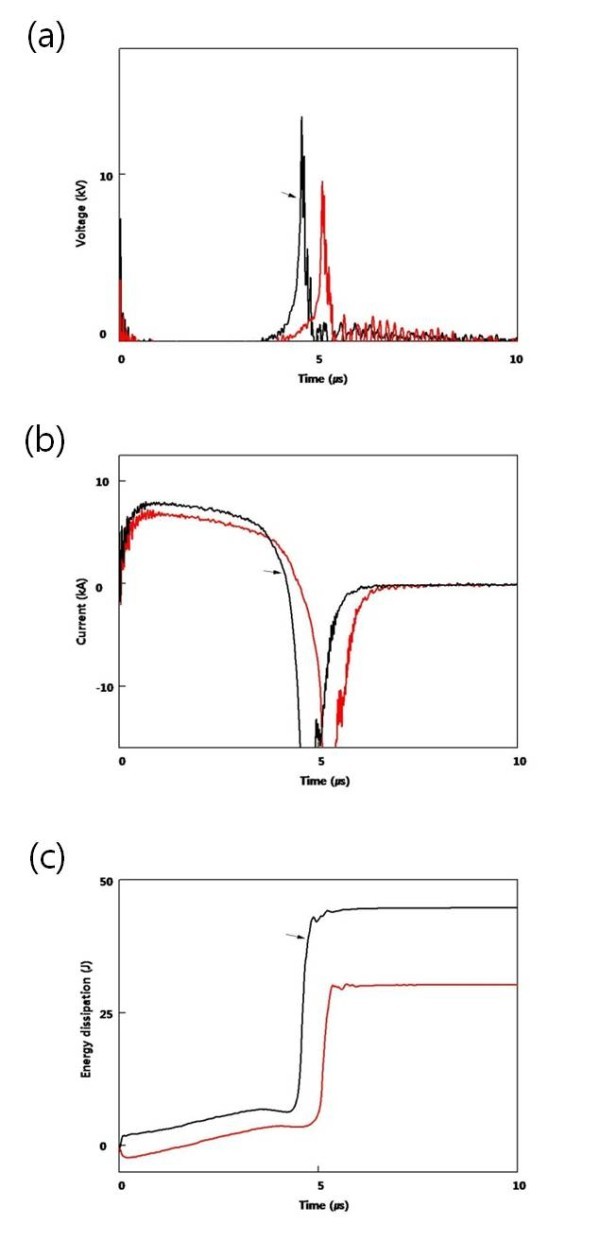
**Voltage and current waveforms under various capacitances**: Voltage (**a**), current (**b**), and deposited energy (**c**) as a function of time (*black line*, 30 μF; *red line*, 7.5 μF).

Figure 4 shows the voltage and current waveforms under various capacitances. Under the same discharge voltage, the pulsed voltage was faster and higher with 30 μF than with 7.5 μF. The increase in capacitance resulted in a slight increase in current and energy deposition.

**Figure 5:**
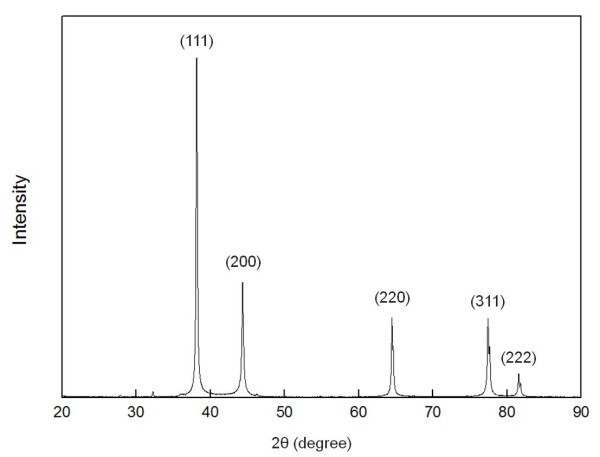
**XRD pattern of Ag nanoparticles produced by EEWL**.

**Figure 6:**
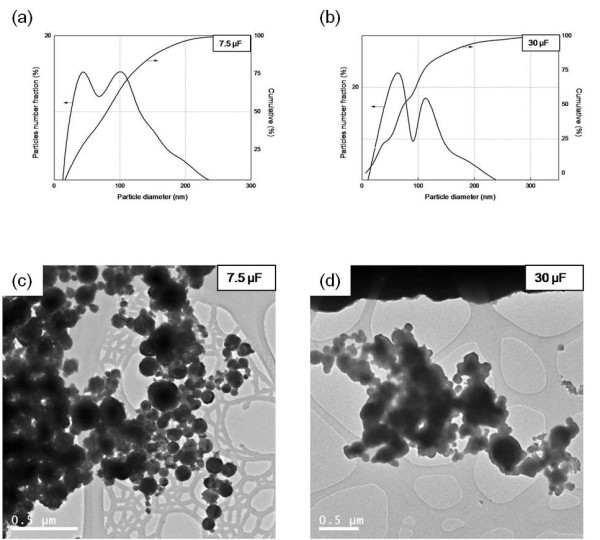
**Size distribution and morphology of Ag nanoparticles under different capacitances**: 7.5 μF (**a**, **c**), 30 μF (**b**, **d**).

The energy deposited in the wire affected the size and shape of the particles (Table 3). As the energy deposited in the wire increased, the particle size decreased. Figure 5 shows the X-ray diffraction [XRD] patterns of silver nanoparticles generated through the EEWL. Only a silver peak was observed. The average size of the synthesized Ag nanoparticles calculated from the XRD patterns by Scherrer's equation is around 23.9 nm. Figure 6 shows the size distribution from TEM images and the morphology of the Ag nanoparticles at different capacitances. The charging time and duration of plasma can influence the formation of particles. The Ag particles were spherical in shape and the particle distribution approached a bimodal distribution, which may have resulted from liquid droplet formation-induced aggregation of particles [17].

**Table 3:** Average particle size and zeta potential of Ag nanofluids produced by EEWL in deionized water

Capacitance (μF)	30	7.5
Average particle size (nm)	123.5	1,283
Zeta potential (mV)	-37	-11.80

Figure 6b shows the coagulation of Ag nanoparticles, which was likely a result of three potential processes. First, agglomeration may have occurred during handling or processing due to the high specific surface area of the nanoparticles. Second, it may have occurred during the drying process of sample preparation. Finally, it may have occurred during condensation.

**Figure 7:**
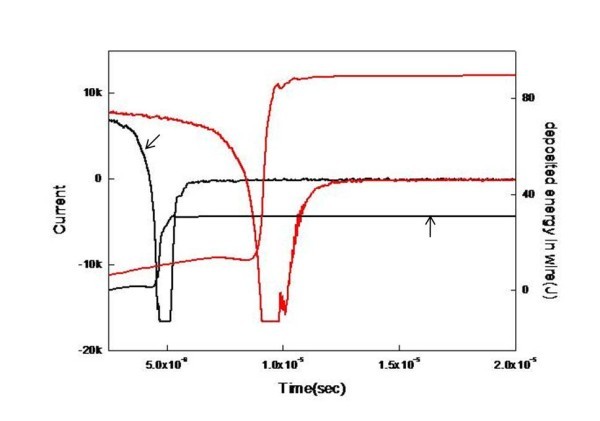
**Effect of liquid type on current and deposited energy in the wire**. Ethanol (*black line*), water (*red line*)

Figure 7 shows the effect of liquid type on the current and energy. Deionized water resulted in smaller particle sizes and good stability (Table 4). The plasma expanded in the medium due to the temperature difference between the plasma and the medium. A low viscosity led to a larger expansion in the plasma volume; thus, the particle size was smaller.

**Table 4:** Average particle size and zeta potential of Ag nanofluids

	Deionized water	Ethanol
Average particle size (nm)	123.5	1,873
Zeta potential (mV)	-37	1.43

**Figure 8:**
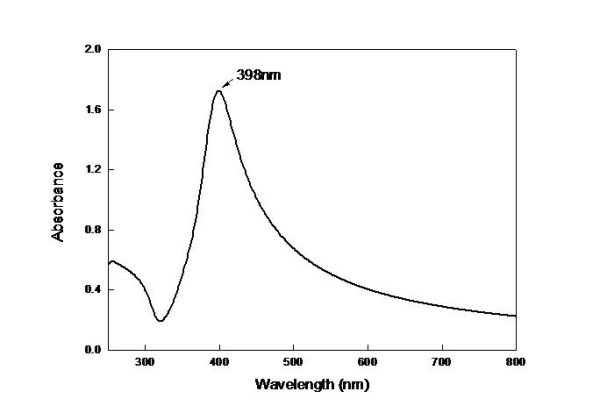
**UV/Vis absorption spectra of Ag nanofluids**.

Defects were observed in the particle structure. Under non-equilibrium process conditions, the synthesized particles were imperfect and had defects, such as twins and dislocations. The UV/Vis absorption spectra of Ag nanofluids are shown in Figure 8. Maximum absorbance occurred at ~398 nm, similar to previous reports [18].

**Figure 9:**
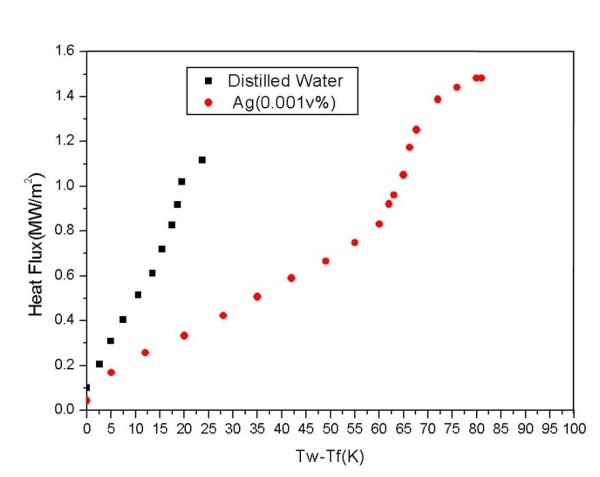
**Boiling curves for pure water and 0.001 vol.% Ag nanofluids**.

**Figure 10:**
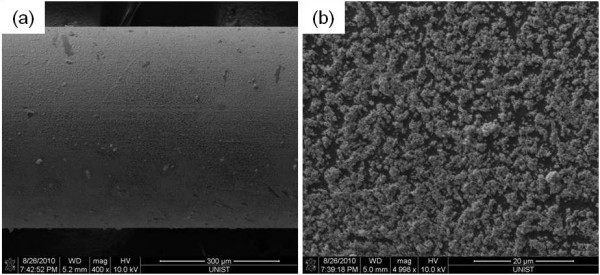
**Ag particles coating the surface of the wire after boiling**. Magnification: ×400 (**a**), ×4,998 (**b**).

Typical boiling curves for pure water and Ag nanofluids are shown in Figure 9. The boiling curves of Ag nanofluids showed no significant change due to the very low concentration. The increase in the CHF can be explained by the changes in surface properties [19]. Analysis of the wire surface showed that a silver layer built up during nanofluids boiling, as shown in Figure 10.

### Process optimization

DOE was used to compare the discharge features and to optimize process parameters. EEWL is a function of many parameters, including wire diameter, length and material, electrical circuit properties, and ambient conditions. In this experiment, there were four parameters of interest that affected the size and dispersibility of particles: capacitance, wire length, liquid volume, and liquid type. Each parameter had two levels or values. The experimental parameters are summarized in Table 5. The experiments were set up using Minitab. The DOE used a 1/4 fractional factorial design of the full factorial design with two repetitions. This resulted in eight total experiments, which was less than the full factorial design of 16, as shown in Table 5. Designs with factors that are usually set at two levels assume that the effects of the factors are linear. Generally, a factor is only evaluated at the low and high points. Ideally, there should be one or more center points to improve the reliability. Further analysis can compare the measurements of the dependent variable at the center point with the average values of the design.

**Table 5:** Experimental conditions

	Capacitance (μF)	Length (mm)	Liquid volume (mL)	Liquid
Run 1	30	38	1,000	Ethanol
Run 2	7.5	38	1,000	Deionized water
Run 3	7.5	38	500	Ethanol
Run 4	30	28	500	Ethanol
Run 5	7.5	28	500	Deionized water
Run 6	30	28	1,000	Deionized water
Run 7	7.5	28	1,000	Ethanol
Run 8	30	38	500	Deionized water
Run 9	15	33	500	Deionized water
Run 10	15	33	1,000	Deionized water
Run 11	15	33	500	Ethanol
Run 12	15	33	1,000	Ethanol

**Figure 11:**
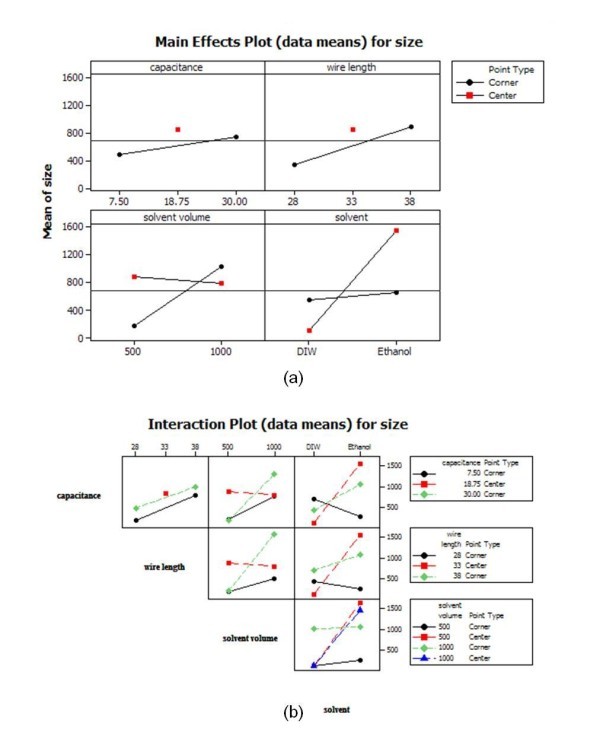
**Quantitative assessment of the effects of the parameters of EEWL for particle size**. Main effects (**a**) and interaction plots (**b**).

**Figure 12:**
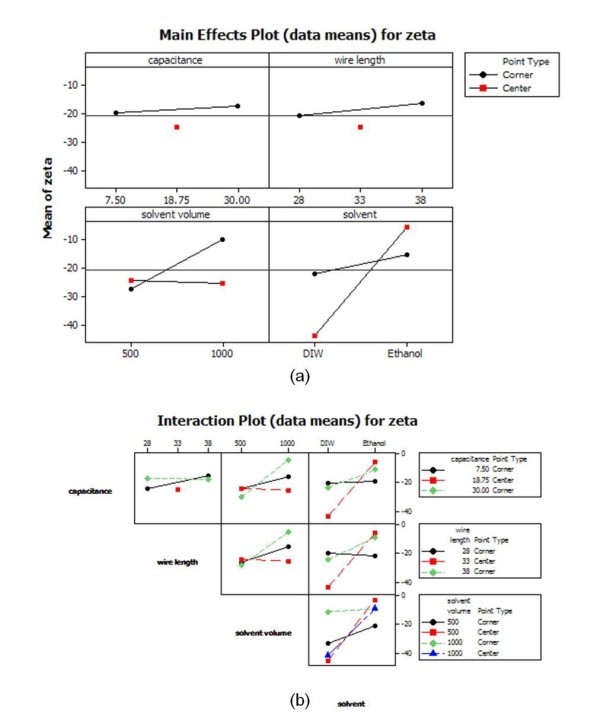
**Quantitative assessment of the effects of the parameters of EEWL for particle zeta potential**. Main effects (**a**) and Interaction plots (**b**)

**Figure 13:**
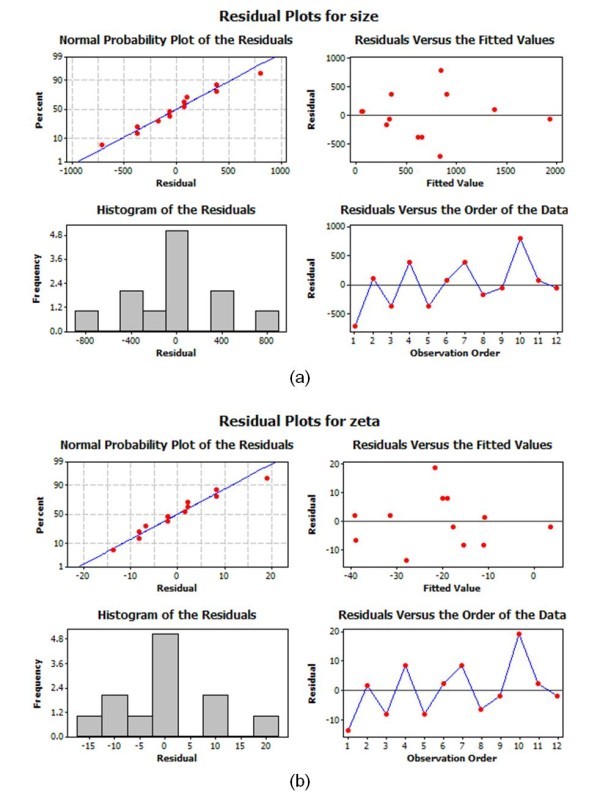
**Residual plots for Ag nanofluids**. Particle size (**a**) and zeta potential (**b**).

Figures 11 and 12 show the main effects plot, interaction plot, and cube plot for size and zeta potential to quantitatively assess the effects of the parameters of EEWL. In the cube plot, the mean responses of the factors are displayed on the corners of the cube. Low levels of the factors are to the left, front, or bottom and high levels to the right, back, or front of the cube. Figure 13 represents the residual plot for size and zeta potential. The normal probability plot did not show any unusual features.

**Figure 14:**
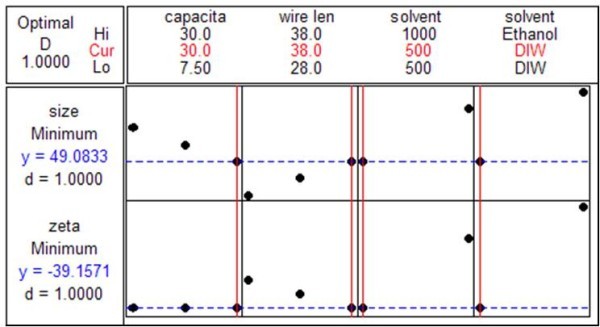
**Response optimization conditions**.

The process was optimized when capacitance was 30 μF, wire length was 38 mm, liquid volume was 500 mL, and liquid type was deionized water. These conditions corresponded to a particle size of 49 nm and zeta potential of -39.15 mV, as shown in Figure 14. These predicted optimized parameters were confirmed by repeating the experiment, and the results for particle size and zeta potential were 118.9 nm and -42.5 mV, respectively. There were differences between the prediction and the experimental results. However, the experimental results showed the smallest and most stable Ag nanoparticles.

## Conclusions

In this study, we produced Ag nanofluids by electrical explosion of wires in liquids. By optimizing control parameters, we decreased the particle size under fast explosion conditions and long plasma duration with low-viscosity media. Low viscosity decreased the particle size and dispersion stability due to greater expansion in the plasma volume. The process was optimized by ROT when capacitance was 30 μF, wire length was 38 mm, liquid volume was 500 mL, and the liquid type was deionized water. For the repeated experiment, the average particle size of the Ag nanoparticles in water was 118.9 nm and the zeta potential was -42.5 mV. The CHF of the 0.001-vol.% Ag nanofluid was higher than that of pure water.

## Competing interests

The authors declare that they have no competing interests.

## Authors' contributions

EJP and HWP designed and guided all aspects of this work. SWL participated in the experiments. ICB participated in the design of the experiments.
